# Vulnerable conditions syndemic, depression, and suicidal ideation among school children in China: cross-sectional census findings

**DOI:** 10.1186/s13034-024-00751-x

**Published:** 2024-05-23

**Authors:** Hanqian Wang, Jingjing Lu, Honghui Zhao, Lu Li, Xudong Zhou

**Affiliations:** 1 Institute of Social Medicine, School of Medicine, Zhejiang University, 866 Yuhangtang Road, 310058 Hangzhou, Zhejiang Province China; 2 Zhejiang Shuren University, Hangzhou, China; 3 The Second Affiliated Hospital, Zhejiang University School of Medicine, Hangzhou, Zhejiang China

**Keywords:** Syndemic, Children, Vulnerable conditions, Depression, Suicidal ideation

## Abstract

**Background:**

Mental health issues (depression and suicidal ideation) are increasingly common in children and emerge as escalating public health concerns. The syndemics that underline the importance of risk factor clustering provides a framework for intervention, but there is a lack of research on syndemics involving the adverse interactions of children’s mental health problems. This study therefore examined the cumulative and synergistic effects of vulnerable conditions on depression and suicidal ideation among children in China.

**Methods:**

A mental health screening census of students in grades 5–12 was conducted from November 2022 to January 2023 in Nanling County, Anhui Province, China. The prevalence and co-occurrence of vulnerable conditions (unfavorable parental marital status, left-behind experience, bullying victimization, and self-harm behavior), depression, and suicidal ideation and the cumulative and synergistic effects of vulnerable conditions on depression and suicidal ideation were explored.

**Results:**

Nearly a quarter of students (24.8%) reported at least two syndemic conditions. Overall, the prevalence of depression and suicidal ideation were 20.2% and 24.2% respectively. The odds of depression and suicidal ideation were higher for children with one or more vulnerable conditions and were ten times higher for children with three or more vulnerable conditions compared with those without any vulnerable condition. These four vulnerable conditions can increase the odds of depression and suicidal ideation by interacting synergistically with each other.

**Conclusion:**

Our findings signal the importance of addressing mental health syndemics among children in China by simultaneously considering concurrent vulnerable conditions.

**Supplementary Information:**

The online version contains supplementary material available at 10.1186/s13034-024-00751-x.

## Introduction

Globally, suicide is one of the leading causes of death in children and adolescents [1]. Research on depression and suicidal intention in children has received growing attention worldwide. As noted, 17.2% of Chinese children aged 6–15 reported depressive symptoms [2], and 32% reported suicidal ideation [3]. Depression serves as a trigger for suicidal behavior [4] with suicidal ideation lying on a continuum of suicidal behaviors [5] and being a potential precursor to suicide [6].

Children exposed to some vulnerable conditions, including bullying victimization [7, 8], self-harm behavior [9], parental divorce [2], and left-behind experience [10] (“left-behind children” refers to children whose fathers or mothers or both parents have migrated to another city outside of their original residence area for six months or more, and also means long-term parental absences [11], reflecting a phenomenon deeply rooted in China’s population mobility and urbanization.), face a higher risk of depression and suicidal ideation. As separate risk factors of mental health, these vulnerable conditions have been well-examined in previous studies. However, these vulnerable conditions may co-occur and have syndemic effects on depression and suicidal ideation in children. Accordingly, research focusing on one specific vulnerable condition rather than multiple vulnerable conditions may underestimate the full strength of the combination of various vulnerable conditions on health outcomes (i.e., depression and suicide). Additionally, constricted attention to a single vulnerable condition may lead to an overestimation of its impact, which may actually have been a result of other co-occurring vulnerable conditions.

The Syndemic theory is a framework for understanding how two or more co-occurring vulnerable conditions can interact synergistically within a specific population and social context to mutually increase the burden of adverse health outcomes [12, 13]. With the concept of syndemics well applied to HIV research, such as the SAVA syndemic [14], more attention is being paid to its role in mental health. One study on youth in California [15] found that the syndemic effects of multiple adverse mental health conditions significantly increased the risk of depression. Another survey in the U.S. noted the syndemic effects of recent maltreatment including emotional maltreatment, neglect, and witnessing family violence on young people’s suicidal or self-harm ideation [16]. Syndemic approaches have also been adopted in studies on children in China, such as exploring the effect of varied types of family violence on children [17], the risk of multiple psychosocial problems on substance use [18], and the clusters of adverse psychosocial syndemic conditions on sexual health risks among adolescent girls [19]. However, global research on child syndemics has a narrow focus on substance use, sexual health risks, child victims, and maltreatment risks, barely touching the severe issues of depression and suicidal ideation in children. To date, understanding of syndemics among children’s mental health is still limited, with most studies conducted among adults.

Syndemic theory posits that mental health issues stem not from singular causes, but from the interplay of multiple factors [13]. Depression and suicidal ideation among children represent intricate social and public health challenges influenced by various psychological, physiological, and social factors [1]. These critical risk factors are not isolated but rather intricately interconnected, synergistically contributing to mental health outcomes. Embracing syndemic approaches offers fresh perspectives to unravel the complex multifactorial underpinnings of depression and suicidal ideation in children. Syndemics may heighten the vulnerability of children to depression and suicide, while risk factors associated with these mental health conditions can also foster the development of syndemic states. Childhood depression and suicide can stem from various factors [2, 7–10], including bullying, self-harm behavior, parental divorce, and left behind experiences, all of which may elevate the risk of depression and suicide. These multiple factors can interact and mutually influence each other, exacerbating children’s depression and suicidal ideation. Addressing these factors individually may prove less effective, and tackling them collectively may yield a more significant impact on children’s mental health problems. Existing evidence primarily focuses on the effects of each influencing factor on suicidal ideation and depression, with limited exploration of the cumulative and synergistic impacts of multiple factors. Therefore, it is instructive to introduce the Syndmeic theory to analyze the cumulative and synergistic effects of multiple factors on suicidal ideation and depression among children. This study initiates by delineating syndemic conditions, characterized by four vulnerability factors: unfavorable parental marital status, left-behind experience, bullying victimization, and self-harm behavior. Leveraging syndemics as a conceptual framework to comprehend these conditions and their intricate interconnections, may facilitate the design of more effective interventions aimed at reducing the incidence of childhood depression and suicide. An extensive investigation into the syndemic of mental health issues among children holds promise in elucidating potential risk and protective factors. Such insights can facilitate early detection and intervention for mental health concerns, thereby enabling timely measures to address emerging issues. Moreover, this endeavor may foster the refinement of screening tools for mental health problems, guiding the development of individualized and specialized interventions tailored to children’s specific needs. Targeted support systems within families, schools, and communities can also strengthened to enhance children’s mental resilience and adaptability, thereby effectively mitigating the incidence of mental health problems. Additionally, insights gleaned from this approach can inform service delivery strategies geared towards enhancing mental health outcomes among children.

**Fig. 1:**
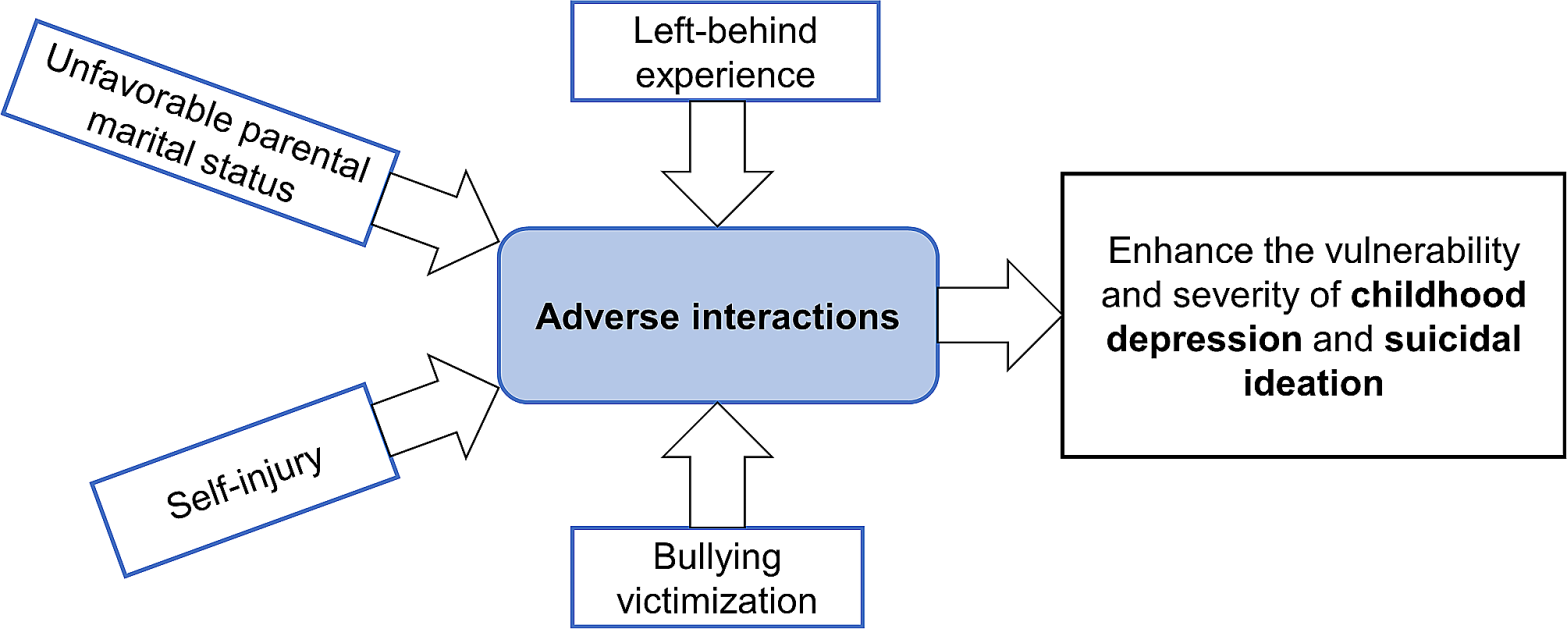
Model of a syndemic of unfavorable parental marital status, left-behind experience, bullying victimization, and self-harm behavior and associations with child depression and suicidal ideation

Thus, to better study the effects of vulnerable syndemic conditions on children, the present study aims to recognize the syndemic of unfavorable parental marital status, left-behind experience, bullying victimization, and self-harm behavior, which is aligned with the syndemic conceptual model [13] (Fig. 1: Model of a syndemic of unfavorable parental marital status, left-behind experience, bullying victimization, and self-harm behavior and associations with child depression and suicidal ideation). Applying syndemic framework to assess synergistically interacting mental health problems, this study aimed to understand whether unfavorable parental marital status, left-behind experience, bullying victimization, and self-harm behavior co-occur, and to assess the cumulative and synergistic effects of these variables on depression and suicide ideation in children. Specifically, we posit that (1) unfavorable parental marital status, left-behind experience, bullying victimization, self-harm behavior, childhood depression, and suicidal ideation co-occur; (2) exposure to vulnerable conditions (unfavorable parental marital status, left-behind experience, bullying victimization, and self-harm behavior) is associated with increased risks of depression and suicidal ideation; (3) unfavorable parental marital status, left-behind experience, bullying victimization, and self-harm behavior interact to increase the likelihood of depression and suicidal ideation.

## Method

This is a mental health screening census of students in grades 5–12 covering all 35 primary schools, 27 middle schools, and 6 high schools in Nanling County, Anhui Province conducted between November 2022 and January 2023. Every school was included and none were excluded. Anhui is a province in the southern central region of China with a population of over 61 million, and with the 8th highest GDP among Chinese provinces. First, the county’s education department issued a notice to all educational institutions in October 2022, informing parents that their children would undergo a mental health screening at school unless they objected. Here, students with known abnormal mental development, mental illness, or physical disability diagnosed by doctors during the survey period were excluded, as they had difficulty independently completing the questionnaire. Students whose parents did not provide approval for the informed consent form were also excluded. Second, all eligible students completed the mental health screening questionnaire in the school computer room under the supervision of a teacher. No personal information of students or parents, including those who did not participate in the survey, was recorded. Across all the schools in Nanling, 30,386 (5th-12th grade; aged 11–17) out of 33,820 eligible students completed the screening questionnaire, representing a response rate of 89.85%. The study was approved by the ethics committee of the School of Public Health, Zhejiang University (ZGL202108-1).

## Measures

### Socio-demographic variables

In this study, socio-demographic characteristics included: gender, grade, family economic status, paternal and maternal education level (primary school or below/middle school/high school or above), unfavorable parental marital status (divorced, remarried, one or both of them died/married), and left-behind experience. The left-behind experience was assessed by the following questions: “Has your father ever migrated to another place for six months or more ever since you were born?” and “Has your mother ever migrated to another place for six months or more ever since you were born?” Children reporting “Yes, currently migrating.” or “Yes, ever migrated.” to either question were labeled as “yes”, otherwise were labeled as “no”.

### Bullying victimization

Students’ bullying victimization experience was measured with the first item of the Chinese version of the Olweus Bully/Victim Questionnaire (OBVQ). This self-reported item was used to assess whether students had generally experienced bullying. If students experienced any type of victimization at least two to three times a month, they were classified as victims (yes), otherwise they were classified as “no”.

### Self-harm behavior

Self-harm behavior was assessed by the following question: “Have you ever deliberately hurt yourself in the past year (Such as cutting, scratching, jumping from a height, taking excessive medication, and swallowing indigestible substances, etc.)?” This variable was then dichotomized into “yes” (at least once) and “no”.

### Depression and suicidal ideation

We adopt the Patient Health Questionnaire-9 (PHQ-9), a standardized tool with established reliability and validity, to assess student depression. The PHQ-9 contains nine questions corresponding to the nine DSM-IV symptoms for major depressive disorders (MDD) during the past two weeks [20]. The answer categories were based on a 4-point response scale, with the categories “not at all” (0), “several days” (1), “more than half of the days” (2), and “nearly every day” (3). As such, total scores range from 0 to 27, with scores ≥ 10 denoting clinically significant depressive symptoms [21]. Suicidal ideation was assessed through PHQ-9 item 9 (“thoughts that you would be better off dead or of hurting yourself in some way”) [21]. Students who reported “not at all” were coded as having “no suicidal ideation” and all other responses were coded as “have suicidal ideation”. The Cronbach’s alpha in the present study was 0.883.

### Statistical analysis

Descriptive analyses reported frequencies and percentages of all variables. We used chi-square tests to measure the associations between socio-demographics and the two mental health outcomes (depression and suicidal ideation). In this calculation, we identified the co-occurrence of these vulnerable conditions using the additive approach commonly used to test syndemics theory. We also performed a sensitivity analysis to assess the effect of excluding missing participants on overall results. Before conducting the cumulative effects analysis, we tested sets of multivariable logistic regression models to identify the interrelatedness of unfavorable parental marital status, left-behind experience, bullying victimization, self-harm behavior, depression, and suicidal ideation for the participants.

Two multivariable logistic regression models were conducted to estimate the association between each variable and the two outcomes. Another two multivariable logistic regression models were conducted to identify the cumulative effect of unfavorable parental marital status, left-behind experience, bullying victimization, and self-harm behavior on depression and suicidal ideation. To estimate the accumulative effect, each logistic regression analysis was adjusted for socio-demographic variables, including gender, grade, family economic status, paternal education, and maternal education. Following established methodologies for syndemic-related studies [14, 22], for each outcome, Model 1 included solely sociodemographic characteristics. In Model 2, we integrated sociodemographic characteristics with additional factors, namely unfavorable parental marital status, left-behind experience, bullying victimization, and self-harm behavior. Model 3 encompassed both sociodemographic characteristics and syndemic conditions.

Finally, we tested synergistic interactions by evaluating interactions using multivariable logistic regression models, and adjusted for socio-demographic variables. For each outcome, we included variables representing two-way interactions between unfavorable parental marital status, left-behind experience, bullying victimization, and self-harm behavior. We also included variables representing three-way interactions among these four variables.

Analyses of all models were adjusted for gender, grade, family economic status, paternal education level, and maternal education level for socio-demographic factors associated with depression [4] and suicidal ideation [3, 9]. These were available from the questionnaire of this mental health screening census. All statistical analyses were performed using SPSS (IBM Corp. Released 2012. IBM SPSS Statistics for Windows, Version 21.0. Armonk, NY: IBM Corp.) and assumed a statistical significance level of *p* < 0.05.

## Results

After excluding 409 participants with missing data, participants with a complete depression variable (29,977 of 30,386; 98.7%) were included in the final analysis. We found the results showing no significant changes in most cases, before and after exclusion (Additional file 1 Sensitivity analysis on the effect of included and censored the excluding participants on the overall results).

### Socio-demographic characteristics

Of the 29,977 students (Table 1), more than half were male (53.1%), and with a medium family economic level (63.2%). Nearly half (42.6%) of the students were in grades 7–9. Most of the students reported paternal (61.2%) and maternal (56.5%) education levels to be “middle school”. 15.6% of students reported their parents divorced, remarried, or died; 65.2% reported left-behind experience; 8.2% reported bullying victimization experience; and 17.0% reported self-harm behavior.

**Table 1:** Sociodemographic characteristics and mental health conditions with depression and suicidal ideation (*N* = 29,977)

Variables	Full sample *N* (%)	Depression		*p*	Suicidal ideation		*p*
		<10	≥ 10		No	Yes	
*Gender*				< 0.001			< 0.001
Male	15,914 (53.1)	13,309 (55.7)	2605 (42.9)		12,840 (56.5)	3074 (42.5)	
Female	14,063 (46.9)	10,601 (44.3)	3462 (57.1)		9896 (43.5)	4167 (57.5)	
*Grade*				< 0.001			< 0.001
5–6	6969 (23.2)	6082 (25.4)	887 (14.6)		5473 (24.1)	1496 (20.7)	
7–9	12,760 (42.6)	10,051 (42.0)	2709 (44.7)		9413 (41.4)	3347 (46.2)	
10–12	10,248 (34.2)	7777 (32.5)	2471 (40.7)		7850 (34.5)	2398 (33.1)	
*Family economic status(quintiles)*				< 0.001			< 0.001
1	670 (2.4)	435 (1.9)	235 (4.1)		435 (2.0)	235 (3.5)	
2	3366 (12.0)	2357 (10.6)	1009 (17.7)		2296 (10.8)	1070 (15.8)	
3	17,696 (63.2)	14,367 (64.4)	3329 (58.5)		13,668 (64.3)	4028 (59.7)	
4	5491 (19.6)	4502 (20.2)	989 (17.4)		4240 (19.9)	1251 (18.5)	
5	790 (2.8)	661 (3.0)	129 (2.3)		622 (2.9)	168 (2.5)	
*Paternal education level*				< 0.001			0.009
Primary school or below	4457 (15.3)	3455 (14.8)	1002 (17.1)		3310 (14.9)	1147 (16.3)	
Middle school	17,859 (61.2)	14,343 (61.6)	3516 (59.9)		13,649 (61.6)	4210 (59.9)	
High school or above	6857 (23.5)	5505 (23.6)	1352 (23.0)		5190 (23.4)	1667 (23.7)	
*Maternal education level*				< 0.001			0.027
Primary school or below	6425 (22.3)	5010 (21.8)	1415 (24.5)		4829 (22.0)	1596 (23.1)	
Middle school	16,283 (56.5)	13,121 (57.0)	3162 (54.8)		12,480 (57.0)	3803 (55.1)	
High school or above	6092 (21.2)	4894 (21.3)	1198 (20.7)		4595 (21.0)	1497 (21.7)	
*Parental marital status*				< 0.001			< 0.001
Married	25,302 (84.4)	20,515 (85.8)	4787 (78.9)		19,561 (86.0)	5741 (79.3)	
Divorced, remarried, One or both of them died	4675 (15.6)	3395 (14.3)	1280 (21.1)		3175 (14.0)	1500 (20.7)	
*Left-behind experience*				< 0.001			< 0.001
No	10,445 (34.8)	8564 (35.8)	1881 (31.0)		8114 (35.7)	2331 (32.2)	
Yes	19,532 (65.2)	15,346 (64.2)	4186 (69.0)		14,622 (64.3)	4910 (67.8)	
*Bullying victimization*				< 0.001			< 0.001
No	27,208 (91.8)	22,257 (94.2)	4951 (82.3)		21,243 (94.5)	5965 (83.1)	
Yes	2441 (8.2)	1376 (5.8)	1065 (17.7)		1227 (5.5)	1214 (16.9)	
*Self-harm behavior*				< 0.001			< 0.001
No	24,877 (83.0)	21,627 (90.5)	3250 (53.6)		21,152 (93.0)	3725 (51.4)	
Yes	5100 (17.0)	2283 (9.5)	2817 (46.4)		1584 (7.0)	3516 (48.6)	
*Syndemic condition*				< 0.001			< 0.001
No syndemic condition	7314 (24.7)	6561 (27.8)	753 (12.5)		6393 (28.5)	921 (12.8)	
One syndemic condition	14,990 (50.6)	12,642 (53.5)	2348 (39.0)		12,257 (54.5)	2733 (38.1)	
Two syndemic conditions	5761 (19.4)	3806 (16.1)	1955 (32.5)		3359 (14.9)	2402 (33.5)	
Three syndemic conditions	1414 (4.8)	581 (2.5)	833 (13.8)		431 (1.9)	983 (13.7)	
Four syndemic conditions	170 (0.6)	43 (0.2)	127 (2.1)		30 (0.1)	140 (2.0)	

### Depression and suicidal ideation

Among all the students (Table 1), 20.2% scored more than 9 points in depression, and 24.2% reported suicidal ideation. Higher depression scores and suicidal ideation are associated with a higher proportion of parents divorced, remarried, or died, and more experiences of being left behind, being bullied, or engaging in self-harm behavior (ps < 0.05).

### Cooccurrence of unfavorable parental marital status, left-behind experience, bullying victimization, and self-harm behavior

Overall (Table 1), more than three-quarters of the students (75.3%) reported at least one of the four syndemic conditions, namely unfavorable parental marital status, left-behind experience, bullying victimization, or self-harm behavior. Nearly a quarter of students (24.8%) reported at least two syndemic conditions. Students with high depression scores or suicidal ideation reported significantly more types of syndemic conditions (ps < 0.05).

### Multivariable logistic regression analyses among intersecting health problems

Table 2 presents the multivariable logistic regression models of the four vulnerable conditions with depression and suicidal ideation. Left-behind experience (aOR = 1.65; 95% CI = 1.52–1.79), being bullied (aOR = 1.32; 95% CI = 1.17–1.49), self-harm behavior (aOR = 1.18; 95% CI = 1.07–1.31), depression (aOR = 1.20; 95% CI = 1.09–1.33), and suicidal ideation (aOR = 1.20; 95% CI = 1.08–1.32) were significantly associated with higher odds of students’ parents divorced, remarried, or died.

**Table 2:** Multivariable logistic regression analyses of unfavorable parental marital status, left-behind experience, bullying victimization, self-harm behavior, depression, and suicidal ideation (*N* = 29,977)

Independent variables	Unfavorable parental marital status (aOR, 95% CI)	Left-behind experience (aOR, 95% CI)	Bullying victimization (aOR, 95% CI)	Self-harm behavior (aOR, 95% CI)	Depression (aOR, 95% CI)	Suicidal ideation (aOR, 95% CI)
Unfavorable parental marital status	/	1.65 (1.52–1.79)***	1.34 (1.19–1.51)***	1.19 (1.07–1.31)**	1.20 (1.09–1.33)***	1.19 (1.08–1.32)***
Left-behind experience	1.65 (1.52–1.79)***	/	1.02 (0.92–1.13)	1.06 (0.98–1.15)	1.14 (1.05–1.24)**	1.07 (0.99–1.15)
Bullying victimization	1.32 (1.17–1.49)***	1.02 (0.92–1.13)	/	1.96 (1.74–2.19)***	2.44 (2.16–2.75)***	1.93 (1.71–2.17)***
Self-harm behavior	1.18 (1.07–1.31)**	1.06 (0.98–1.15)	1.90 (1.69–2.13)***	/	2.92 (2.67–3.18)***	6.80 (6.25–7.40)***
Depression	1.20 (1.09–1.33)**	1.14 (1.05–1.23)*	2.37 (2.10–2.67)***	2.89 (2.65–3.15)***	/	9.68 (8.95–10.47)***
Suicidal ideation	1.20 (1.08–1.32)***	1.07 (0.99–1.15)	1.96 (1.73–2.21)***	6.80 (6.25–7.40)***	9.70 (8.97–10.49)***	/

Adjusting for socio-demographics, including gender, grade, family economic status, paternal education, and maternal education

*p < 0.05, **p < 0.01, ***p < 0.001

aOR, adjusted OR

The odds of parents divorced, remarried, or died (aOR = 1.65; 95% CI = 1.52–1.79), and depression (aOR = 1.14; 95% CI = 1.05–1.23) were significantly associated with higher odds of left-behind experience.

Higher odds of experiencing bullying were significantly associated with having divorced, remarried, or died parents (aOR = 1.34; 95% CI = 1.19–1.51), having self-harm behavior (aOR = 1.90; 95% CI = 1.69–2.13), depression (aOR = 2.37; 95%CI = 2.10–2.67), and suicidal ideation (aOR = 1.96; 95% CI = 1.73–2.21).

The experience of being bullied (aOR = 1.96; 95% CI = 1.74–2.19), parents divorced, remarried, or died (aOR = 1.19; 95% CI = 1.07–1.31), depression (aOR = 2.89; 95% CI = 2.65–3.15), and suicidal ideation (aOR = 6.80; 95% CI = 6.25–7.40) were significantly associated with higher odds of self-harm behavior.

Experience of divorced, remarried, or died parents (aOR = 1.20; 95% CI = 1.09–1.33), being left-behind (aOR = 1.14; 95% CI = 1.05–1.24), being bullied (aOR = 2.44; 95% CI = 2.16–2.75), self-harm behavior (aOR = 2.92; 95% CI = 2.67–3.18), and suicidal ideation (aOR = 9.70; 95% CI = 8.97–10.49) were significantly associated with higher odds of depression.

Experiencing parents divorced, remarried, or died (aOR = 1.19; 95% CI = 1.08–1.32), being bullied (aOR = 1.93; 95% CI = 1.71–2.17), having self-harm behavior (aOR = 6.80; 95% CI = 6.25–7.40), and depression (aOR = 9.68; 95% CI = 8.95–10.47) were significantly associated with higher odds of suicidal ideation.

### Cumulative effects of unfavorable parental marital status, left-behind experience, bullying victimization, and self-harm behavior on depression and suicidal ideation

Table 3 shows the results of multivariable logistic regression models of the association between each vulnerable condition and the two outcome measures, and the cumulative effects of all vulnerable conditions on depression and suicidal ideation. There was a significant trend in the relationship between the number of syndemic conditions ranging from 0 to 4 and the proportions of depression and suicidal ideation, such that as every one-unit increase in the number of syndemic conditions, the odds of being depressed and having suicidal ideation increased significantly. Specifically, compared to students without any vulnerable conditions, those with one, two, three, or four syndemic conditions were significantly more likely to suffer from depression: (aOR = 1.61; 95% CI = 1.74–1.77), (aOR = 4.68; 95% CI = 4.24–5.18), (aOR = 13.98; 95% CI = 12.08–16.19), (aOR = 29.68; 95% CI = 19.77–44.56). Similarly, compared to students without any vulnerable conditions, those with one, two, three, or four syndemic conditions were significantly more likely to have suicidal ideation: (aOR = 1.60; 95% CI = 1.47–1.75), (aOR = 5.30; 95% CI = 4.83–5.82), (aOR = 15.87; 95% CI = 13.71–18.38), (aOR = 30.46; 95% CI = 19.64–47.25).

**Table 3:** Multivariable logistic regression models of cumulative effects of four syndemic factors on depression and suicidal ideation

Variables	Depression			Suicidal ideation		
	Model 1	Model 2	Model 3	Model 1	Model 2	Model 3
*Gender*						
Male	Ref	Ref	Ref	Ref	Ref	Ref
Female	1.73 (1.63–1.84)***	1.58 (1.48–1.70)***	1.74 (1.63–1.85)***	1.80(1.70–1.91)***	1.64 (1.54–1.75)***	1.84 (1.73–1.96)***
*Grade*						
5–6	Ref	Ref	Ref	Ref	Ref	Ref
7–9	1.88 (1.72–2.05)***	2.13 (1.93–2.35)***	2.04 (1.86–2.25)***	1.33(1.23–1.43)***	1.37 (1.25–1.50)***	1.39 (1.28–1.51)***
10–12	2.09 (1.91–2.29)***	2.97 (2.68–3.30)***	2.64 (2.39–2.91)***	1.09(1.01–1.18)*	1.34 (1.22–1.47)***	1.28 (1.18–1.40)***
*Family economic status(quintiles)*						
1	Ref	Ref	Ref	Ref	Ref	Ref
2	0.74 (0.61–0.89)**	0.76 (0.62–0.93)**	0.77 (0.63–0.93)**	0.82(0.68–0.98)*	0.85 (0.69–1.06)	0.87 (0.71–1.06)
3	0.40 (0.34–0.47)***	0.48 (0.40–0.58)***	0.50 (0.41–0.60)***	0.49(0.42–0.59)***	0.62 (0.51–0.75)***	0.64 (0.53–0.77)***
4	0.40 (0.33–0.48)***	0.46 (0.38–0.57)***	0.50 (0.41–0.61)***	0.49(0.41–0.59)***	0.59 (0.48–0.73)***	0.65 (0.53–0.79)***
5	0.42 (0.33–0.55)***	0.45 (0.34–0.60)***	0.49 (0.37–0.64)***	0.49(0.38–0.62)***	0.52 (0.40–0.69)***	0.57 (0.44–0.75)***
*Father education level*						
Primary school or below	Ref	Ref	Ref	Ref	Ref	Ref
Middle school	0.95 (0.86–1.04)	0.96 (0.87–1.06)	0.96 (0.87–1.06)	0.95(0.87–1.04)	0.98 (0.88–1.08)	0.97 (0.89–1.07)
High school or above	1.01 (0.89–1.13)	1.02 (0.90–1.16)	1.10 (0.97–1.17)	1.01(0.91–1.13)	1.03 (0.91–1.17)	1.12 (0.99–1.26)
*Mother education level*						
Primary school or below	Ref	Ref	Ref	Ref	Ref	Ref
Middle school	0.97 (0.89–1.05)	0.96 (0.88–1.05)	0.95 (0.87–1.03)	0.97(0.90–1.04)	0.96 (0.88–1.05)	0.95 (0.87–1.03)
High school or above	1.06 (0.95–1.19)	1.04 (0.92–1.17)	1.04 (0.93–1.17)	1.08(0.97–1.20)	1.06 (0.94–1.19)	1.06 (0.95–1.18)
*Parental marital status*						
Married		Ref			Ref	
Divorced, remarried, One or both of them died		1.30 (1.19–1.43)***			1.29 (1.18–1.41)***	
*Left-behind experience*						
No		Ref			Ref	
Yes		1.17 (1.09–1.26)**			1.12 (1.05–1.20)**	
*Bullying victimization*						
No		Ref			Ref	
Yes		3.19 (2.86–3.55)***			2.77 (2.49–3.09)***	
*Self-harm behavior*						
No		Ref			Ref	
Yes		7.26 (6.74–7.83)***			10.77 (9.99–11.61)***	
*Syndemic condition*						
No syndemic condition			Ref			Ref
One syndemic condition			1.61 (1.47–1.77)***			1.60 (1.47–1.75)***
Two syndemic conditions			4.68 (4.24–5.18)***			5.30 (4.83–5.82)***
Three syndemic conditions			13.98 (12.08–16.19)***			15.87 (13.71–18.38)***
Four syndemic conditions			29.68 (19.77–44.56)***			30.46 (19.64–47.25)***

*p < 0.05, **p < 0.01, ***p < 0.001

### Model of synergistically interacting epidemics

#### Depression

We used multivariable logistic regression models with product terms to estimate a multiplicative interaction for vulnerable conditions on depression (Table 4).

**Table 4:** Multiplicative two-/three-way interactions of syndemic factors on depression and suicidal ideation

Variables	Model 1^a^	Model 2^a^	Model 3^a^	Model 4^a^	Model 5^a^	Model 6^a^	Model 7^a^	Model 8^a^	Model 9^a^	Model 10^a^	Model 11^a^	Model 12^a^
Two-way interactions												
Outcome: Depression												
UPMS × LBE	1.54 (1.40–1.69)***						0.88 (0.77–0.99)*					
UPMS × BV		4.80 (3.93–5.87)***					1.66 (1.25–2.21)**					
UPMS × SHB			6.97 (6.01–8.09)***				2.22 (1.83–2.68)***					
LBE × BV				4.23 (3.77–4.74)***			1.53 (1.30–1.81)***					
LBE × SHB					7.05 (6.49–7.66)***		4.84 (4.41–5.30)***					
BV × SHB						12.45 (10.62–14.59)***	3.65 (2.99–4.46)***					
**Three-way interactions**												
UPMS × LBE × BV								4.74 (3.77–5.97)***				1.18 (0.83–1.69)
UPMS × LBE × SHB									6.79(5.73–8.04)***			4.83 (4.02–5.79)***
UPMS ×BV × SHB										13.94 (9.81–19.81)***		1.52 (0.91–2.55)
LBE × BV × SHB											11.87 (9.83–14.34)***	8.96 (7.33–10.96)***
**Outcome: Suicidal ideation**												
**Two-way interactions**												
UPMS × LBE	1.47 (1.34–1.60)***						0.84 (0.74–0.94)**					
UPMS × BV		4.05 (3.33–4.94)***					1.52 (1.14–2.03)**					
UPMS × SHB			8.61 (7.34–10.09)***				2.40 (1.97–2.93)***					
LBE × BV				3.91 (3.50–4.37)***			1.52 (1.29–1.78)***					
LBE × SHB					9.87 (9.06–10.75)***		6.96 (6.34–7.64)***					
BV × SHB						15.20 (12.75–18.13)***	4.47(3.62–5.52)***					
**Three-way interactions**												
UPMS × LBE × BV								3.92 (3.13–4.92)***				1.09 (0.78–1.54)
UPMS × LBE × SHB									8.45 (7.05–10.13)***			6.35 (5.25–7.68)***
PMS ×BV × SHB										13.02 (8.95–18.92)***		1.60 (0.94–2.72)
LBE × BV × SHB											14.41 (11.69–17.78)***	11.25 (9.05–13.98)***

Adjusting for socio-demographics, including gender, grade, family economic status, paternal education, and maternal education

*p < 0.05, **p < 0.01, ***p < 0.001

^a^ Model 1: unfavorable parental marital status × left-behind experience; Model 2: unfavorable parental marital status × bullying victimization; Model 3: unfavorable parental marital status × self-harm behavior; Model 4: left-behind experience × bullying victimization; Model 5: left-behind experience × self-harm behavior; Model 6: bullying victimization × self-harm behavior; Model 7: all two-way terms; Model 8: unfavorable parental marital status × left-behind experience × bullying victimization; Model 9: unfavorable parental marital status × left-behind experience × self-harm behavior; Model 10: unfavorable parental marital status × bullying victimization × self-harm behavior; Model 11: left-behind experience × bullying victimization × self-harm behavior; Model 12: all three-way terms

Unfavorable parental marital status = UPMS; Left-behind experience = LBE; Bullying victimization = BV; Self-harm behavior = SHB

We estimated a multiplicative interaction between every two vulnerable conditions. In the two-way interactions, the joint effects of unfavorable parental marital status with left-behind experience (model 1: aOR = 1.54, 95% CI: 1.40–1.69), unfavorable parental marital status with bullying victimization (model 2: aOR = 4.80, 95% CI: 3.93–5.87), unfavorable parental marital status with self-harm behavior (model 3: aOR = 6.97, 95% CI: 6.01–8.09), left-behind experience with bullying victimization (model 4: aOR = 4.23, 95% CI: 3.77–4.74), left-behind experience with self-harm behavior (model 5: aOR = 7.05, 95% CI: 6.49–7.66), and bullying victimization with self-harm behavior (model 6: aOR = 12.45, 95% CI: 10.62–14.59) were associated with depression.

In the logistic regression model including all two-way product terms and adjusting for gender, grade, family economic status, and paternal/maternal education level (model 7 in Table 4), we found multiplicative interactions for joint effects of unfavorable parental marital status with bullying victimization (aOR = 1.66, 95% CI: 1.25–2.21), unfavorable parental marital status and self-harm behavior (aOR = 2.22, 95% CI: 1.83–2.68), left-behind experience and bullying victimization (aOR = 1.53, 95% CI:1.30–1.81), left-behind experience and self-harm behavior (aOR = 4.84, 95% CI: 4.41–5.30), and bullying victimization and self-harm behavior (aOR = 3.65, 95% CI: 2.99–4.46) on depression. We found that the joint effects of unfavorable parental marital status and left-behind experience acted as a protective factor (aOR = 0.88, 95% CI: 0.77–0.99) on depression.

In sum, by examining synergistic effects, we found that any two vulnerable conditions - unfavorable parental marital status and left-behind experience, unfavorable parental marital status and bullying victimization, unfavorable parental marital status and self-harm behavior, left-behind experience and bullying victimization, left-behind experience and self-harm behavior, bullying victimization and self-harm behavior - were associated with depression when they co-occurred.

In the three-way interactions, the joint effects of unfavorable parental marital status with left-behind experience with bullying victimization (model 8: aOR = 4.74, 95% CI: 3.77–5.97), unfavorable parental marital status with left-behind experience with self-harm behavior (model 9: aOR = 6.79, 95% CI: 5.73–8.04), unfavorable parental marital status with bullying victimization with self-harm behavior (model 10: aOR = 13.94, 95% CI: 9.81–19.81), and left-behind experience with bullying victimization with self-harm behavior (model 11: aOR = 11.87, 95%CI: 9.83–14.34) were associated with depression.

In a regression model that includes all three-way product terms, we found multiplicative interaction for joint effects of unfavorable parental marital status and left-behind experience and self-harm behavior (aOR = 4.383, 95%CI: 4.02–5.79), and left-behind experience and bullying victimization and self-harm behavior (aOR = 8.96, 95%CI: 7.33–10.96) on depression.

When exploring the synergistic effects of three-way interactions, the interactions between any three vulnerable conditions - unfavorable parental marital status and left-behind experience and bullying victimization, unfavorable parental marital status and left-behind experience and self-harm behavior, unfavorable parental marital status and bullying victimization and self-harm behavior, left-behind experience and bullying victimization and self-harm behavior - were all associated with depression.

### Suicidal ideation

Table 4 shows logistic regression models with product terms to assess the multiplicative effects of vulnerable conditions on suicidal ideation.

We found the joint effects of unfavorable parental marital status with left-behind experience (model 1: aOR = 1.47, 95% CI: 1.34–1.60), unfavorable parental marital status with bullying victimization (model 2: aOR = 4.05, 95% CI: 3.33–4.94), unfavorable parental marital status with self-harm behavior (model 3: aOR = 8.61, 95% CI: 7.34–10.09), left-behind experience with bullying victimization (model 4: aOR = 3.91, 95% CI: 3.50–4.37), left-behind experience with self-harm behavior (model 5: aOR = 9.87, 95% CI: 9.06–10.75), and bullying victimization with self-harm behavior (model 6: aOR = 15.20, 95% CI: 12.75–18.13) on suicidal ideation.

In a logistic regression model including all two-way product terms and controlling for sociodemographic factors (model 7 in Table 4), we found multiplicative interactions for joint effects of unfavorable parental marital status and bullying victimization (aOR = 1.52, 95% CI: 1.14–2.03), unfavorable parental marital status and self-harm behavior (aOR = 2.40, 95% CI: 1.97–2.93), left-behind experience and bullying victimization (aOR = 1.52, 95% CI: 1.29–1.78), left-behind experience and self-harm behavior (aOR = 6.96, 95% CI: 6.34–7.64), and bullying victimization and self-harm behavior (aOR = 4.47, 95% CI: 3.62–5.52) on suicidal ideation. The joint effects of unfavorable parental marital status and left-behind experience acted as a protective factor (aOR = 0.84, 95% CI: 0.74–0.94) on suicidal ideation.

The joint effects of unfavorable parental marital status with left-behind experience with bullying victimization (model 8: aOR = 3.92, 95% CI: 3.13–4.92), unfavorable parental marital status with left-behind experience with self-harm behavior (model 9: aOR = 8.45, 95% CI: 7.05–10.13), unfavorable parental marital status with bullying victimization with self-harm behavior (model 10: aOR = 13.02, 95% CI: 8.95–18.92), and left-behind experience with bullying victimization with self-harm behavior (model 11: aOR = 14.41, 95% CI: 11.69–17.78) were associated with suicidal ideation.

In model 12, We found the joint effects of unfavorable parental marital status and left-behind experience and self-harm behavior (aOR = 6.35, 95% CI: 5.25–7.68), and left-behind experience and bullying victimization and self-harm behavior (aOR = 11.25, 95% CI: 9.05–13.98) on suicidal ideation, adjusting for sociodemographic factors.

Overall, two-way interactions and three-way interactions with suicidal ideation were found to have the same results as depression in this study.

## Discussion

This study examined the cumulative and synergistic effects of unfavorable parental marital status, left-behind experience, bullying victimization, and self-harm behavior on depression and suicidal ideation in children. As hypothesized, unfavorable parental marital status, left-behind experience, bullying victimization, self-harm behavior, childhood depression, and suicidal ideation co-occurred. Compared to children without any vulnerable condition, those who experienced one or more of these vulnerable conditions had greater proportions of depression and suicidal ideation, and those who experienced three or more of these conditions had more than ten times higher odds of depression and suicidal ideation. These four vulnerable conditions can increase the odds of depression and suicidal ideation by interacting synergistically with each other. Together, these findings suggest that applying the syndemic approach contributed to understanding the synergistic effects of vulnerable conditions in elevating children’s mental health in schools.

### The co-occurrence of vulnerable conditions syndemic

Our study identifies the concurrent occurrence of unfavorable parental marital status, left-behind experience, bullying victimization, and self-harm behavior within this sample of children in China. Unfavorable parental marital status may precipitate emotional instability, a lack of support, and a diminished sense of security for children over prolonged periods [23]. Consequently, this unstable familial environment may impede children’s development, thereby exacerbating their mental health issues. Moreover, left-behind experience entails prolonged separation between children and their parents [23, 24]. This sense of isolation and emotional detachment may heighten the vulnerability of children to bullying. Children subjected to bullying may also endure protracted negative emotions such as fear, shame, and anger, predisposing them to suicidal ideation or self-harm behavior [25]. Likewise, self-harm behavior in children may stem from unstable family environments, prolonged separation, and inner pain and despair [26], potentially intensifying their depressive symptoms and heightening the likelihood of suicidal ideation.

### The cumulative effects of vulnerable conditions syndemic on depression and suicidal ideation

Unfavorable parental marital status, left-behind experience, bullying victimization, and self-harm behavior were independently related to depression and suicidal ideation; concurrently, the risk of depression and suicidal ideation increased multiply for every additional vulnerable condition added. When all vulnerable conditions co-occurred, the odds of depression were more than 20 times higher and the odds of suicidal ideation were even more than 30 times higher. This is consistent with prior research on left-behind children in China [27], which documented that former or current left-behind children with parental divorced or separated had higher risks of psychological problems. It also aligned with a previous study documenting that the co-occurrence of self-harm behaviors and bullying victimization posed a greater risk of depression [28]. As explored in other populations in China [29], psychosocial factors can coexist and increase the burden of health consequences, which also suggested a syndemic effect. Yet, research on the syndemic effects of mental health problems was mainly focused on the population affected by HIV, indicating a statistically significant positive dose-response relationship between the number of syndemic conditions and HIV-related risks [22, 30, 31].

### The synergistic effects of vulnerable conditions syndemic on depression and suicidal ideation

Moreover, besides the direct cumulative effect, our study revealed synergistic interactions among the four vulnerable conditions and depression and suicidal ideation. The risk of depression and suicidal ideation was significantly higher when two or more vulnerable conditions interacted with each other. Interestingly, we also observed that any vulnerable condition interacting with unfavorable parental marital status and left-behind experience had protective effects on these two outcomes. Children experiencing both unfavorable parental marital status and left-behind experience may exhibit heightened psychological resilience, enabling them to effectively navigate and surmount adversities in their lives [32]. Among all the interactions, the interaction between bullying victimization and self-harm behavior had the greatest effect on depression and suicidal ideation. It is worth noting that there is limited research on the interactive effects of syndemic conditions on depression and suicidal ideation in children. However, previous studies [14] among urban refugee youth have found that intimate partner violence and frequent alcohol use interacted, as did non-partner violence and major depression, to increase the odds of transactional sex engagement. Similarly, research on a sample of men who had sex with men in Taiwan [22] showed the interactive effects of two three-way psychosocial health conditions on HIV infection.

These four vulnerable conditions mutually exacerbate one another, perpetuating a vicious cycle. Unfavorable parental marital status may lead to emotional difficulties in children, heightening their susceptibility to bullying. Victims of bullying may exacerbate their depressive symptoms, rendering them more prone to self-harm or suicidal ideation. Similarly, experiences of being left behind may render children more susceptible to becoming targets of bullying, exacerbated by a lack of family support that hinders their ability to cope with the negative repercussions of bullying. Therefore, it is imperative to underscore the necessity for comprehensive intervention measures that address these vulnerable situations concurrently. Echoing previous calls [13], to fully understand the interactive syndemic impact, further research should analyze the vulnerable conditions rooted at individual and structural levels for children, such as family factors and social environment, that may interact with vulnerable conditions. Findings based on synergistic interactions may provide a much more accurate observation of how clustered vulnerable conditions affect children’s mental health [33], as we identified among children in Nanling in the present study.

### Implication

Our findings suggest that interventions informed by the model of synergistically interacting of these vulnerable conditions suggest that focusing on children with multiple vulnerable conditions concurrently and addressing multiple issues may be more beneficial for children’s mental health than addressing any one issue alone. Child-centered, vulnerable conditions-based, multi-pronged and holistic approaches are needed for mental health promotion among children. Specifically, psychological counseling strategies that are tailored for children with diverse vulnerable conditions, who had a higher prevalence of depression and suicidal ideation in our study, work better to identify vulnerable children early, to provide them with assistance, and to leverage school strengths and supports [34].

We also found that children with depression and suicidal ideation had higher proportions of bullying victimization and self-harm behavior. In view of this, we can take advantage of the support from the school and integrate family guidance and norms to detect and address vulnerable conditions as early as possible to reduce the risk of depression and suicidal ideation. For instance, professional psychological counselors in schools can deliver strategies with mental health promotion approaches that have the potential to improve the mental health and emotional well-being of school children [35, 36], such as resilience-focused interventions, mental health-based lessons, social and emotional learning, and cognitive coping and restructuring skills. Family-delivered support, such as early identification of children’s mental health problems and intimate parent-child connections [37], was instructive to children’s behaviors and assisted the school’s preventive interventions targeted at children’s mental health and vulnerability. However, to date, how to design a comprehensive package containing school engagement and family support that focuses on depression and suicidal ideation prevention for children experiencing vulnerable conditions remains under-explored.

### Limitations

These findings should be viewed in the context of several limitations. First, the cross-sectional design precludes the determination of the direction of causality between the four vulnerable conditions and the two health outcomes. However, our findings did highlight the cumulative and interactive effects of vulnerable conditions on depression and suicidal ideation among children. Future studies could employ longitudinal designs to examine the causal relationships between vulnerable conditions and two health outcomes. Second, all data were collected through self-report and therefore subject to social desirability bias. Social desirability bias, a prevalent concern in research, can substantially influence study outcomes by prompting participants to tailor their responses to conform to societal norms or expectations [38]. Within this study, such bias may lead to the underestimation of mental health issues, as participants may minimize or conceal symptoms of depression or suicidal ideation. Consequently, there exists a risk of underestimating the prevalence or severity of these conditions within the studied population. In this study, participants were offered a relatively independent environment for completing questionnaires, which helped mitigate social expectations from others. Additionally, electronic questionnaires were utilized for data collection, prioritizing anonymity when compared to traditional paper questionnaires. Third, the census results from Nanling County should be extrapolated with caution.

## Conclusion

The cumulative effects and synergistic interactions of parental marital status (divorced, remarried, or died), left-behind experience, bullying victimization, and self-harm behavior are associated with increased risks of depression and suicidal ideation. In order to promote children’s mental health and avoid suicides, the current study suggests that it is crucial to identify children with multiple vulnerable conditions and to address multiple issues concurrently via joint efforts from family and school.

### Electronic supplementary material

Below is the link to the electronic supplementary material.

**Supplementary Material 1:**Sensitivity analysis on the effect of included and censored the excluding participants on the overall results.

## Data Availability

The datasets used and/or analysed during the current study are available from the corresponding author on reasonable request.
