# Supplementary material for: Vulnerable conditions syndemic, depression, and suicidal ideation among school children in China: cross-sectional census findings
Source: Child Adolesc Psychiatry Ment Health. 2024 May 23;18:59. doi: 10.1186/s13034-024-00751-x (PMC11118994; doi:10.1186/s13034-024-00751-x)
Supplement: Supplementary file 1 — Supplementary Material 1:Sensitivity analysis on the effect of included and censored the excluding participants on the overall results. [file 13034_2024_751_MOESM1_ESM.docx]

**Additional file 1** Sensitivity analysis on the effect of included and censored the excluding participants on the overall results

| Variables | N(%) | Censored group | Included group | *P* |
| --- | --- | --- | --- | --- |
| Gender |  |  |  | 0.690 |
| Male | 16127(53.1) | 213(52.1) | 15914(53.1) |  |
| Female | 14259(46.9) | 196(47.9) | 14063(46.9) |  |
| Grade |  |  |  | <0.001 |
| 5-6 | 4512(15.3) | 195(47.7) | 6969(23.2) |  |
| 7-9 | 22236(75.2) | 122(29.8) | 12760(42.6) |  |
| 10-12 | 2826(9.6) | 92(22.5) | 10248(34.2) |  |
| Family economic status(quintiles) |  |  |  | 0.024 |
| 1 | 675(2.4) | 5(2.9) | 670(2.4) |  |
| 2 | 3389(12.0) | 23(13.5) | 3366(12.0) |  |
| 3 | 17817(63.2) | 121(71.2) | 17696(63.2) |  |
| 4 | 5507(19.5) | 16(9.4) | 5491(19.6 |  |
| 5 | 795(2.6) | 5(2.9) | 790(2.8) |  |
| Paternal education |  |  |  | 0.541 |
| Primary school or below | 4512(15.3) | 55(13.7) | 4457(15.3) |  |
| Middle school | 22236(75.2) | 311(77.6) | 21925(75.2) |  |
| High school or above | 2826(9.6) | 35(8.7) | 2791(9.6) |  |
| Maternal education |  |  |  | 0.458 |
| Primary school or below | 6511(22.3) | 86(21.8) | 6425(22.3) |  |
| Middle school | 20158(69.0) | 267(67.8) | 19891(69.1) |  |
| High school or above | 2525(8.6) | 41(10.4) | 2484(8.6) |  |
| Parental marital status |  |  |  | 0.496 |
| Married | 25642(84.4) | 340(83.1) | 25302(84.4) |  |
| Divorced, remarried, One or both of them died | 4744(15.6) | 69(16.9) | 4675(15.6) |  |
| Left-behind experience |  |  |  | 0.530 |
| No | 10581(34.8) | 136(33.3) | 10446(34.8) |  |
| Yes | 19805(65.2) | 273(66.7) | 19532(65.2) |  |
